# The *Bradyrhizobium diazoefficiens* type III effector NopE modulates the regulation of plant hormones towards nodulation in *Vigna radiata*

**DOI:** 10.1038/s41598-021-95925-4

**Published:** 2021-08-16

**Authors:** Pongdet Piromyou, Hien P. Nguyen, Pongpan Songwattana, Pakpoom Boonchuen, Kamonluck Teamtisong, Panlada Tittabutr, Nantakorn Boonkerd, Piyada Alisha Tantasawat, Michael Göttfert, Shin Okazaki, Neung Teaumroong

**Affiliations:** 1 School of Biotechnology, Institute of Agricultural Technology, Suranaree University of Technology, Nakhon Ratchasima, 30000 Thailand; 2 Institute of Global Innovation Research (IGIR), Tokyo University of Agriculture and Technology (TUAT), Fuchu, Tokyo 183-8538 Japan; 3 The Center for Scientific and Technological Equipment, Suranaree University of Technology, Nakhon Ratchasima, 30000 Thailand; 4 School of Crop Production Technology, Institute of Agricultural Technology, Suranaree University of Technology, Nakhon Ratchasima, 30000 Thailand; 5 Institut Für Genetik, Technische Universität Dresden, Helmholtzstrasse 10, 01062 Dresden, Germany; 6 Graduate School of Agriculture, TUAT, Fuchu, Tokyo 183-8509 Japan; 7 Agricultural Research Service (ARS), The U.S. Department of Agriculture (USDA), Beltsville Agricultural Research Center (BARC), Beltsville, MD 20705 USA

**Keywords:** Microbiology, Plant sciences

## Abstract

Host-specific legume-rhizobium symbiosis is strictly controlled by rhizobial type III effectors (T3Es) in some cases. Here, we demonstrated that the symbiosis of *Vigna radiata* (mung bean) with *Bradyrhizobium diazoefficiens* USDA110 is determined by NopE, and this symbiosis is highly dependent on host genotype. NopE specifically triggered incompatibility with *V. radiata* cv. KPS2, but it promoted nodulation in other varieties of *V. radiata*, including KPS1. Interestingly, NopE1 and its paralogue NopE2, which exhibits calcium-dependent autocleavage, yield similar results in modulating KPS1 nodulation. Furthermore, NopE is required for early infection and nodule organogenesis in compatible plants. Evolutionary analysis revealed that NopE is highly conserved among bradyrhizobia and plant-associated endophytic and pathogenic bacteria. Our findings suggest that *V. radiata* and *B. diazoefficiens* USDA110 may use NopE to optimize their symbiotic interactions by reducing phytohormone-mediated ETI-type (PmETI) responses via salicylic acid (SA) biosynthesis suppression.

## Introduction

Symbiosis between rhizobia and legumes initiates by the specific recognition of the two partners involved. In the soil, flavonoids secreted by leguminous plants are the key signalling molecules that are recognized by specific rhizobial species^1^. In general, flavonoids derived from host plants interact with NodD proteins, which leads to the expression of *nod* genes. In several strains, e.g., *Bradyrhizobium diazoefficiens* USDA110, a type three secretion system (T3SS) is also activated. In this case, the T3SS is also involved in nodulation processes by delivering a type three effector (T3E) into the cytosol of eukaryotic host cells^2^. Although many rhizobial T3Es have been identified^3,4^ based on their host genotype-specific symbiotic compatibility or incompatibility with legumes, *Vigna radiata*-T3E relationships have remained limited until now.

Previous studies have shown that *Bradyrhizobium elkanii* USDA61 T3SS was responsible for nodulation restriction in *V. radiata* cv. KPS1 but functioned positively in symbiosis with *V. radiata* cv. CN36^5^ and several *V. mungo* varieties^6^. *B. diazoefficiens* USDA110 is incompatible with *V. radiata* cv. KPS2^7^, but it can effectively nodulate *V. radiata* cv. KPS1 and CN72, mostly due to the presence of a functional T3SS^8^. Among the *B. diazoefficiens* USDA110 T3Es, NopE has been identified as a negative factor specifically responsible for nodulation restriction in KPS2^7^. In this study, we further functionally characterized *B. diazoefficiens* USDA110 T3E NopE in terms of its control of host genotype-specific symbiosis with different *V. radiata* varieties. Symbiotic phenotypes were confirmed by inoculation assays using several selected Thai *V. radiata* varieties. We demonstrated that host genotype-specific nodulation of *V. radiata* is positively/negatively controlled by *B. diazoefficiens* NopE. Interestingly, NopE is required for promoting nodulation in almost all tested *V. radiata* varieties except for KPS2. Furthermore, NopE1 and its paralogue NopE2, exhibiting calcium-dependent autocleavage, yield similar results in modulating KPS1 nodulation and functionally overlap in causing incompatibility with KPS2.

## Results

### Evolutionary and phylogenetic analyses of NopE

To understand the evolutionary history of NopE, we analysed the phylogenetic relationship of NopE homologues among bacterial species. The results demonstrated that NopE was conserved among diverse groups of bacteria (Fig. S1). These bacteria included rhizobia, plant-associated endophytic bacteria, and pathogenic bacteria such as *Xanthomonas*, *Sphingomonas*, and *Candidatus* species. Among the various NopEs, *Bradyrhizobium* NopEs were phylogenetically closely related to their homologues in *Methylobacterium*, the species of which are pink-pigmented, facultatively methylotrophic bacteria (Fig. S1). Most bradyrhizobia were found to have at least one NopE copy, whereas *B. japonicum* and *B. diazoefficiens* strains commonly employed two NopE copies. Impressively, NopE was not identified in nearly any *B. elkanii* bacteria, except for two *B. elkanii* strains, WSM1741 and WSM2783, or in any *Sinorhizobium*, *Ensifer*, *Rhizobium*, and *Mesorhizobium* species (Fig. S1).

### Functional analysis of *B. diazoefficiens* NopEs in determining the nodulation of *V. radiata* varieties

**Figure 1:**
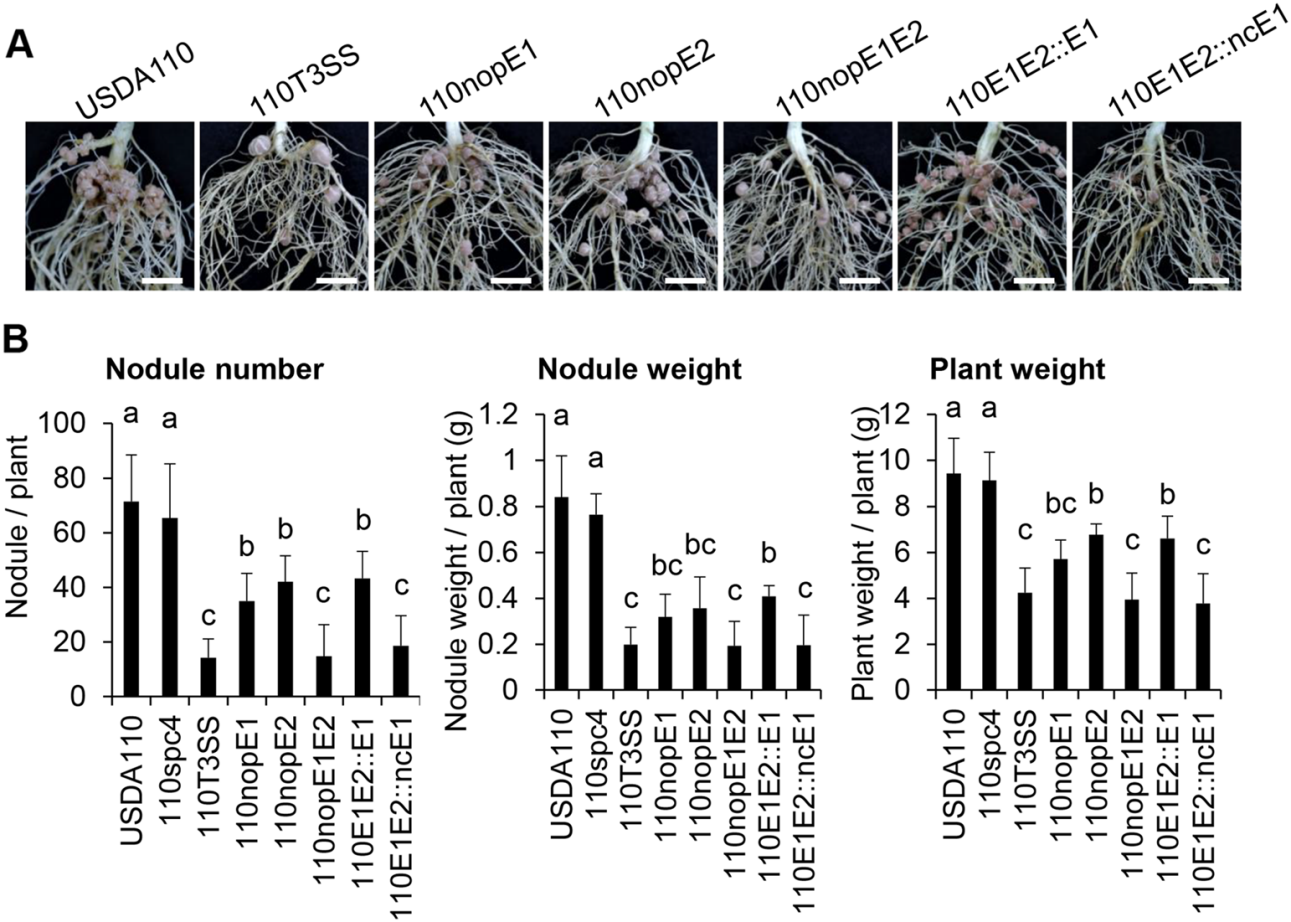
Roots **(A)** and symbiotic properties **(B)** of *Vigna radiata* cv. KPS1 inoculated with *B. diazoefficiens* and its mutant strains. Scale bars: 1 cm. The data shown are the means of at least 9 to 12 plants from two to three independent inoculation assays at 35 dpi. The error bars indicate the standard deviations. The means followed by different letters are significantly different at the 5% level (*P* ≤ 0.05 according to Tukey’s test).

USDA110 T3Es (NopE1 and NopE2) are homologous proteins sharing approximately 77% sequence identity, and both NopEs (NopE1 and NopE2) are also transferred into plant cells. USDA110 NopEs overlap functionally in determining incompatibility with KPS2^7^. The expression levels of blr1806 (*nopE1*) and blr1649 (*nopE2*) were induced by genistein (Fig. S2). We thus hypothesized that *nopE1* and *nopE2* genes are important for *Bradyrhizobium*-*Vigna* symbiosis. To understand the function of NopE in *Vigna* symbiosis, *nopE1* mutant (110nopE1), *nopE2* mutant (110nopE2), and *nopE1*/*nopE2* double mutant (110nopE1E2) were used. The symbiotic characteristics of wild-type USDA110, a T3SS injection mutant strain (110T3SS), and 110E1E2 derivative complementary strains (110E1E2::E1 and 110E1E2::ncE1) were also defined. The 110nopE1E2 greatly reduced nodulation on most Thai *V. radiata* (Table S1). We used *V. radiata* cv. KPS1 to study the biological function of NopE because this cultivar is commonly grown in Thailand. A single mutation in either *nopE1* or *nopE2* strongly reduced nodulation (Fig. 1). 110nopE1E2 showed a similar phenotype to 110T3SS. Furthermore, previous studies demonstrated that calcium-dependent autocleavage activities are required for NopE function in symbiotic incompatibility with KPS2, and the noncleavable NopE1 variant is not active^7^. Our results revealed that complementation of a noncleaved *nopE1* (Table S2) variant into the *nopE1*/*nopE2* mutant (110E1E2::ncE1) background could not enhance KPS1 nodulation to the same degree as that in the wild-type *nopE1* (110E1E2::E1) (Fig. 1).

### Nodulation of *V. radiata* induced by nopE mutants

**Figure 2:**
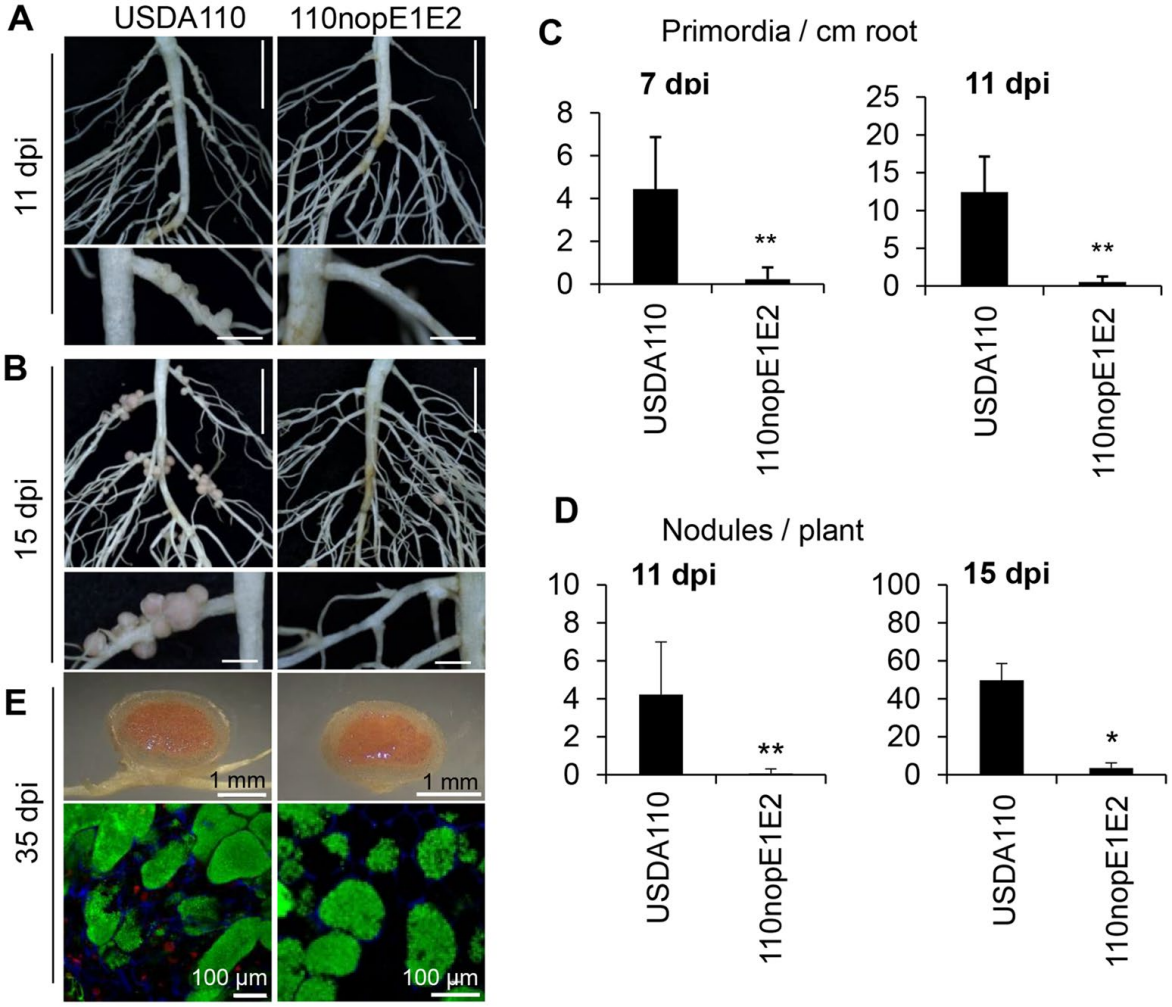
Nodulation properties of *V. radiata* cv. KPS1 inoculated with *B. diazoefficiens* strains. The roots were imaged at 11 **(A)** and 15 **(B)** dpi. **(C)** Nodule primordia per centimetre of root tissue and **(D)** numbers of young nodules per plant observed at 7, 11 and 15 dpi. **(E)** Nodule morphology and propidium iodide (PI)/SYTO9 staining of sectioned nodules. The data shown are the means of at least 6 plants at 7 and 15 dpi and of 18 plants at 11 dpi. The basal regions of lateral roots were sampled (3 samples per plant, n = 18) for microscopy analyses. Scale bars: 1 cm, entire roots; and 2 mm, nodule primordia and young nodules. “*”*P* < 0.05 and “**”*P* < 0.01 according to Student’s *t*-test.

To further explore the pivotal roles of NopE in promoting nodulation, we monitored nodulation-related organogenesis events in KPS1 roots inoculated with USDA110 and 110nopE1E2 at 7, 11, and 15 dpi (Fig. 2). Interestingly, early in the nodulation process, at 7 and 11 dpi, compared with USDA110, 110nopE1E2 caused greatly reduced nodule primordia on taproots and in basal regions of lateral roots (Fig. 2A,C). At 11 and 15 dpi, a few young nodules had formed on the KPS1 roots, although the number was significantly lower than that for USDA110 (Fig. 2B,D). However, nodule morphology and bacteroid differentiation did not differ between USDA110 and 110nopE1E2 inoculation (Fig. 2E).

**Figure 3:**
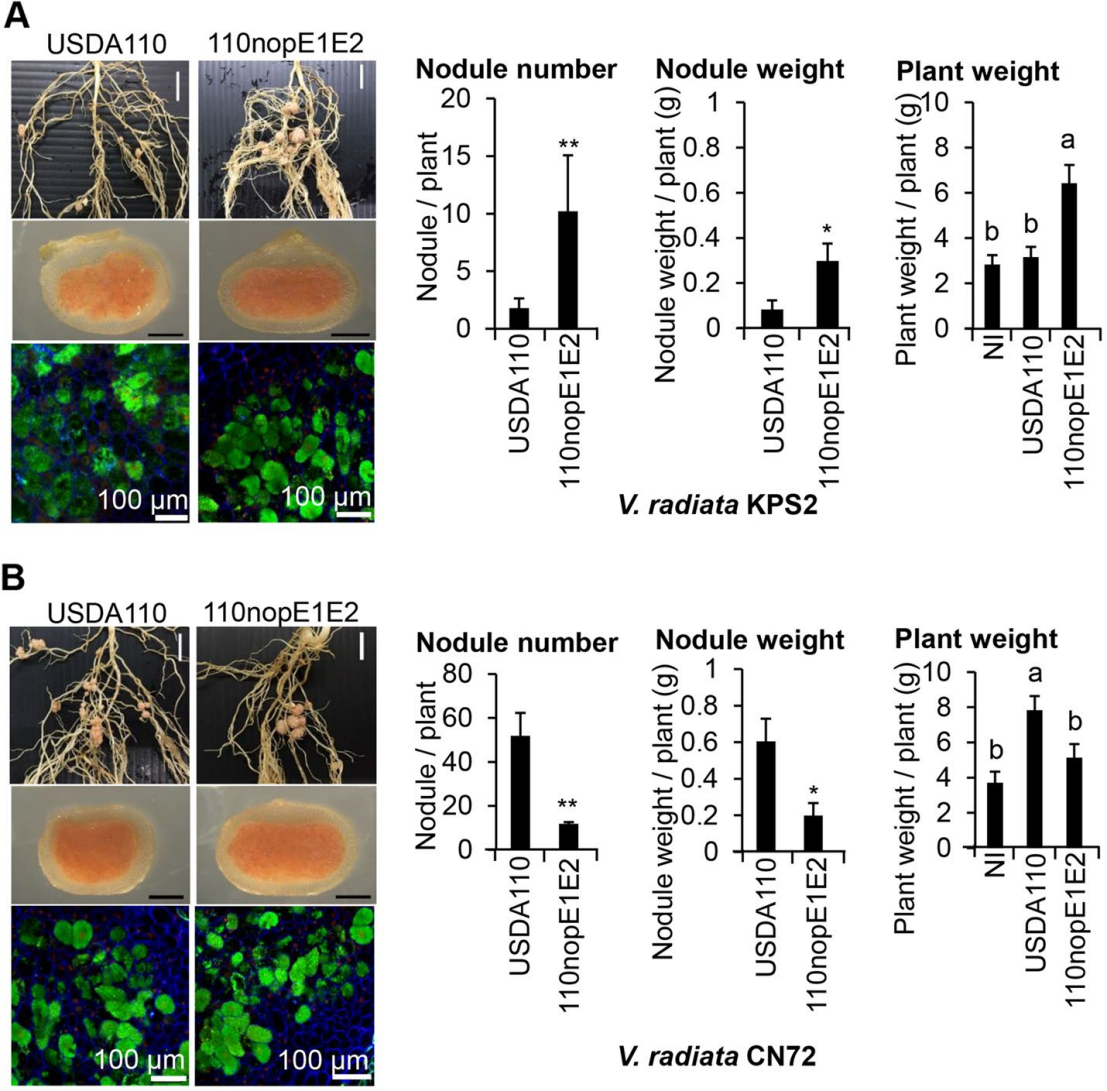
Nodulation properties of *V. radiata* cv. KPS2 **(A)** and its derived strain CN72 **(B)** inoculated with *B. diazoefficiens* strains. The roots were imaged at 35 dpi. The data shown are the means of at least 6 plants. Scale bars: 1 cm, entire roots; 1 mm, nodule morphology; and 100 µm, bacteroid observations with propidium iodide (PI)/SYTO9 staining of sectioned nodules. “*”*P* < 0.05 and “**”*P* < 0.01 according to Student’s *t*-test. The means followed by different letters are significantly different at the 5% level (*P* ≤ 0.05 according to Tukey’s test).

To determine the effect of plant genetic variation on the bradyrhizobial NopE-*V. radiata* interaction, the USDA110 and 110nopE1E2 bradyrhizobial strains were inoculated into both *V. radiata* KPS2 and its derived mutant cultivar CN72 (seeds of KPS2 were irradiated with 600 Gy gamma-rays)^9^. KPS2 growth promotion by 110nopE1E2 inoculation was significantly higher than that by USDA110 inoculation. On the other hand, USDA110 did not significantly promote KPS2 growth compared with that of the noninoculated control (NI) (Fig. 3A). USDA110 caused the formation of a few nodules (approximately 2–4 nodules per plant); however, compared with USDA110, 110nopE1E2 could establish significantly more symbiotic nodules (approximately 15 nodules per plant). For *V. radiata* CN72, compared with 110nopE1E2 inoculation and the NI treatment, the USDA110 inoculation treatment caused significantly more plant growth promotion. For nodule formation, USDA110 could produce up to 50 nodules per plant by 35 dpi (Fig. 3B). 110nopE1E2 could also cause the formation of symbiotic nodules (approximately 5 nodules per plant), although the number of nodules was significantly lower than that of plants inoculated with USDA110. Similarly, nodule morphologies were not different between USDA110 and 110nopE1E2 inoculations, and bacteroid cells were still alive inside both KPS2 and CN72 nodules (Fig. 3).

### *V. radiata* transcriptomic changes in response to the USDA110 nopE effector

**Figure 4:**
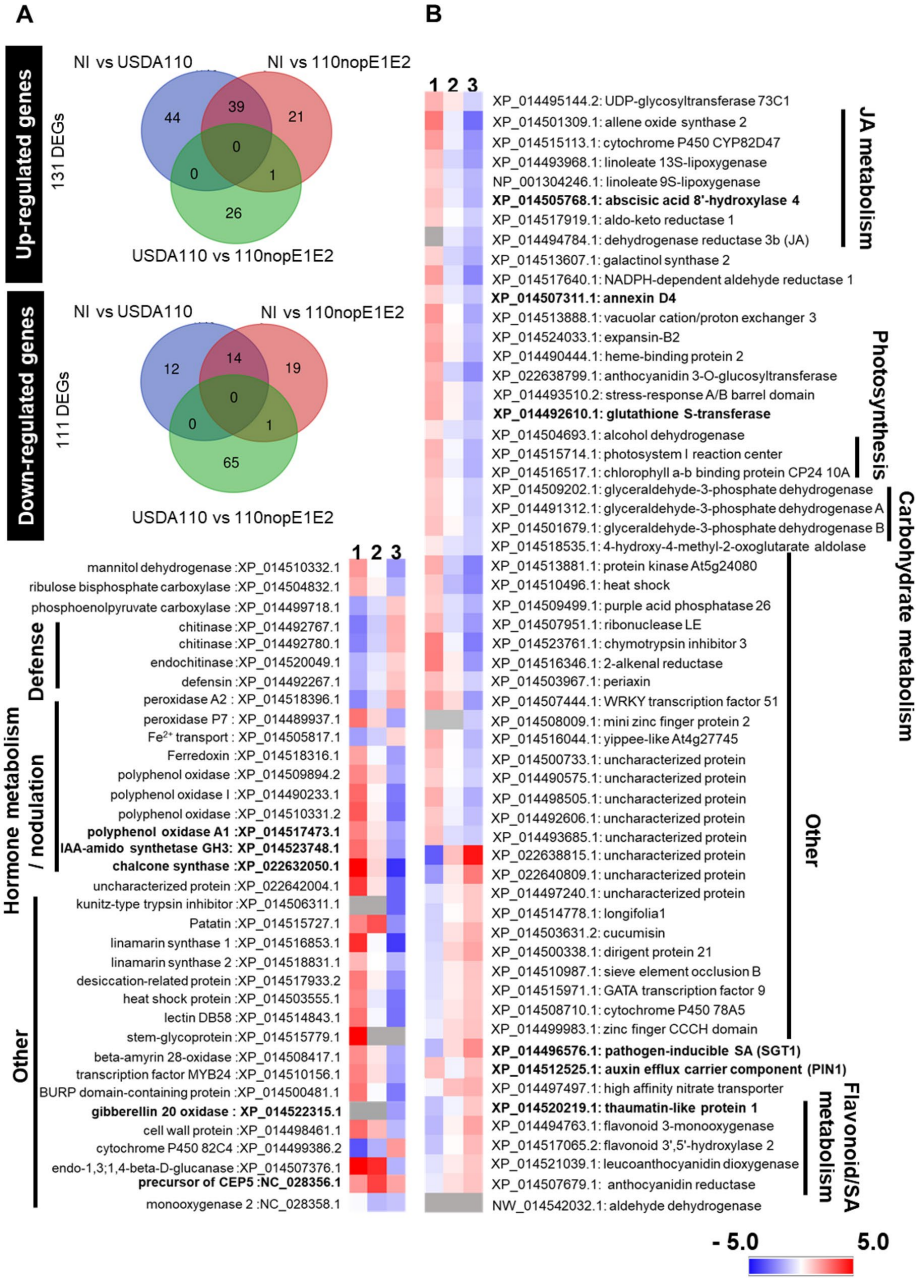
Differentially expressed genes (DEGs) of *Vigna radiata* KPS1 inoculated with USDA110 versus 110nopE1E2. **(A)** Venn diagram showing the number of unique differentially expressed genes in each treatment group or in multiple treatment groups: the noninoculation (NI), wild-type USDA110 inoculation (USDA110), and *nopE* mutant strain inoculation (110nopE1E2) treatment groups. **(B)** The expression patterns of DEGs of USDA110 vs 110nopE1E2 are focused and displayed as the log_2_ fold change (*P*adj < 0.05) of the DEGs. The colour scale bars indicate normalized expression levels of DEGs from NI (1), USDA110 (2), and 110nopE1E2 (3). The heat map was constructed by Microsoft Excel 365.

To determine the RNA-seq transcriptome, total RNA of *V. radiata* KPS1 was extracted and purified from three treatments: NI, USDA110 inoculation, and nopE1E2 inoculation. The main reason for performing the transcriptome analysis was to elucidate the mechanisms involved in the early stage of nodulation by nopE effectors in *Vigna*. Based on RNA-seq transcriptome analysis, several genes were expressed in response to the NI, USDA110, and 110nopE1E2 treatments, and their expression substantially overlapped among the treatments. The differentially expressed genes (DEGs) were analysed using Gene Ontology (GO) functional enrichment analysis for terms involving molecular functions, cellular components and biological processes. The number of DEGs significantly changed (*P* value ≤ 0.05) in response to bacterial inoculation, with the expression of 242 DEGs being significant (Fig. 4A), with 131 upregulated DEGs and 111 downregulated DEGs identified. To more clearly understand the effect of NopE effectors on the *V. radiata* KPS1 response, a comparison of genes differentially expressed between USDA110 and 110nopE1E2 was performed (Fig. 4B). DEGs whose expression was upregulated were associated with jasmonic acid (JA) metabolism, photosynthesis, glutathione S-transferase, carbohydrate metabolic processes, polyphenol oxidase, and others. Interestingly, the genes whose expression was upregulated comprised a small number of mung bean root DEGs annotated as being involved in the JA biosynthetic process, including 2 homologues of linoleate lipoxygenase (*LOX*) and allene oxidase synthase 2 (*AOS2*). The expression of chalcone synthase (*CHS*), which is a key enzyme in the iso-flavonoid biosynthesis pathway (Fig. S3), and genes involved in flavonoid metabolism (Fig. 4B) in mung bean roots was downregulated less in response to USDA110 inoculation than in response to 110nopE1E2 inoculation. *CHS* expression leads to the accumulation of flavonoid and isoflavonoid phytoalexins and is involved in the salicylic acid (SA) defence pathway^10^. We also found decreased levels of pathogen-inducible SA (*SGT1*), thaumatin-like protein 1 and defensin, which are known to be involved in plant immune responses against pathogenic invasion^11,12^. The homologue of the auxin efflux carrier compartment (*PIN1*) gene^13^ was also downregulated in response to USDA110 inoculation. These results suggest that USDA110 also enhances symbiotic interactions with *V. radiata* by impeding host immunity to promote root nodule organogenesis. To confirm the transcriptome data, the expression levels of 12 plant hormone-related genes were verified by qRT-PCR analysis. qRT-PCR was performed for genes associated with GO terms such as ABA 8’ hydroxylase, annexin D4, glutathione S-transferase (*GST*), pathogen-inducible SA (*SGT1*), thaumatin, linoleate 9S-lipoxygenase (*LOX1*), IAA amido synthetase (*GH3*), chalcone synthetase (*CHS*), gibberellin 20 oxidase (*GA20ox*), precursor of CEP5, polyphenol oxidase (*PPO*), and auxin efflux carrier component (*PIN1*) in *V. radiata* KPS1. The expression of these genes significantly differed between the USDA110 and 110nopE1E2 inoculation treatments, the results of which were similar to those of the transcriptome analyses except for *CHS* (Fig. S3).

### Plant hormone-related gene expression

**Figure 5:**
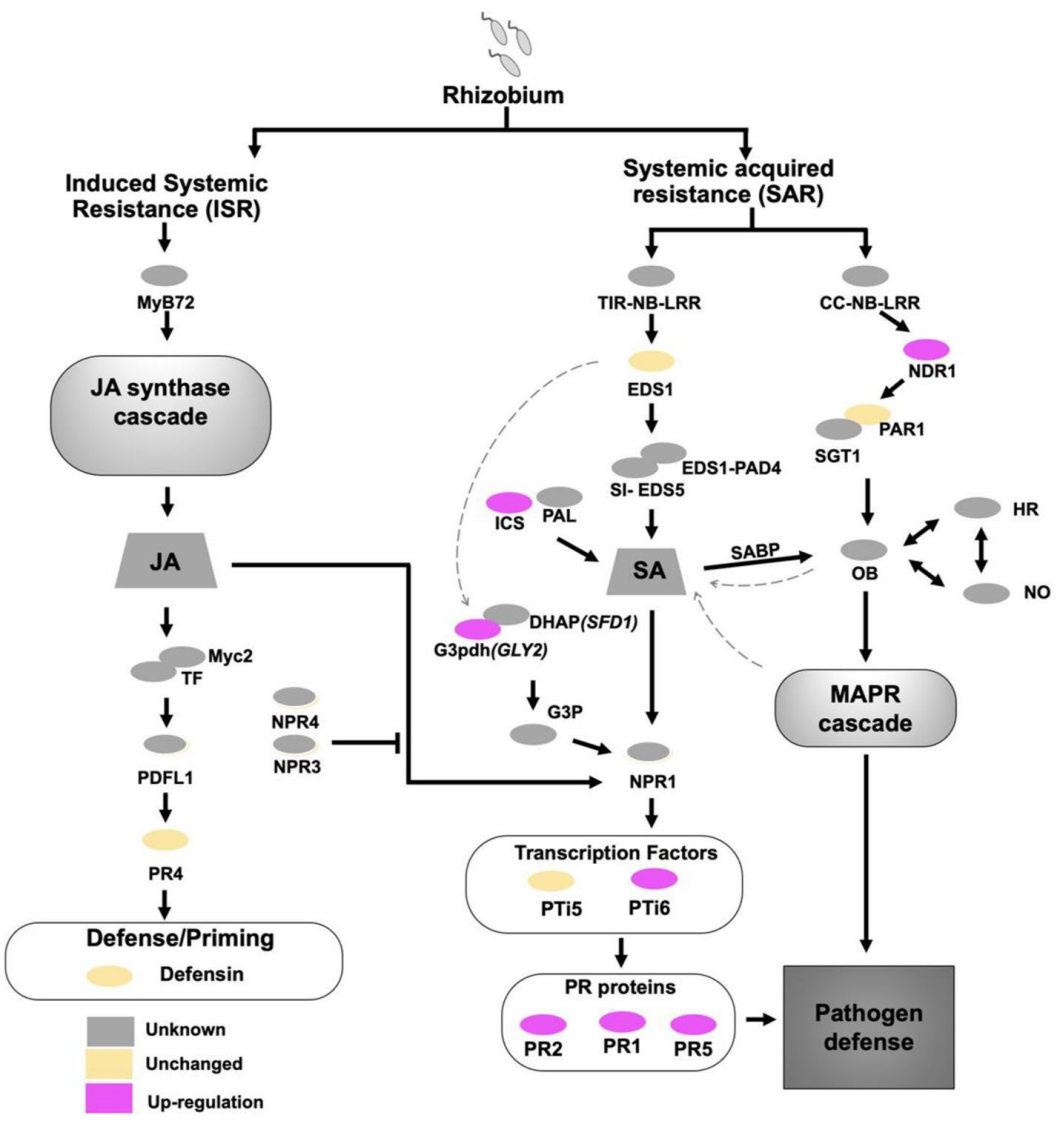
A schematic representation of how salicylic acid triggered the plant immune pathway in *Vigna radiata* cv. KPS1. This representation is based on the gene expression (qRT-PCR) comparison between USDA110 and 110nopE1E2 inoculation: grey colour (unknown), yellow colour (the gene expression was not different between USDA110 and 110nopE1E2 inoculation), and pink colour (the gene expression of 110nopE1E2 inoculation was significantly higher than that of USDA110 inoculation). Significance at *P* < 0.05 is indicated by the means ± standard deviations (n = 3).

**Figure 6:**
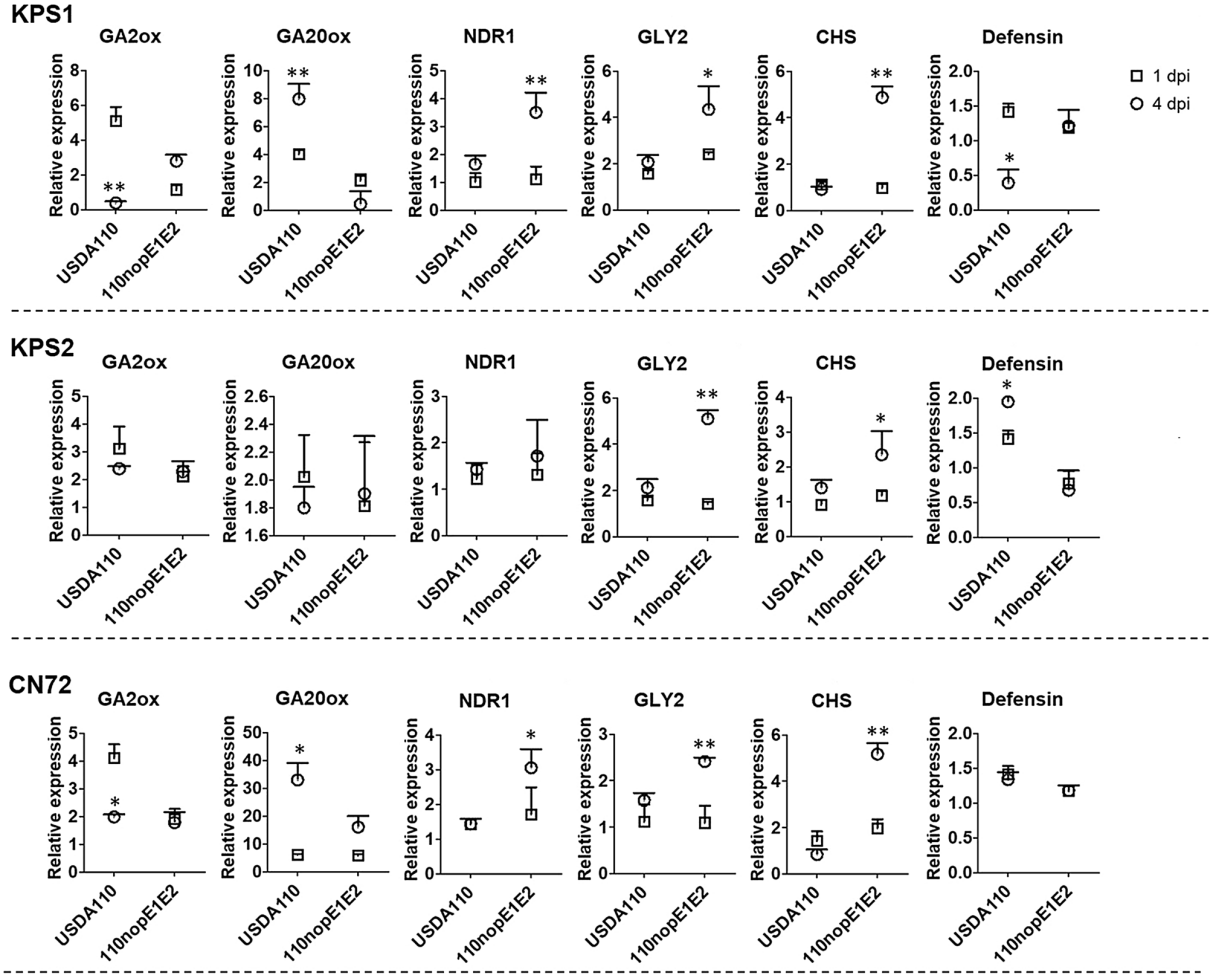
qRT-PCR analysis data for *Vigna radiata* cv. (KPS1, KPS2, and CN72) between USDA110 and 110nopE1E2 inoculations at 1 dip and 4 dpi. The expression levels of genes that were differentially expressed in KPS1, including gibberellin 2 oxidase (*Ga2ox*), gibberellin 20 oxidase (*GA20ox*) nondisease resistance 1 (*NDR1*), glycerol-3-phosphate dehydrogenase (*GLY2*), chalcone synthase (*CHS*), and plant defensin (*PDF*), were determined. Significance at *P* < 0.05, “*”and *P* < 0.01, “**”according to Student’s *t*-test.

The results of our transcriptome analysis and qRT-PCR indicate that NopE may promote USDA110 symbiosis by interfering with *Vigna* hormone signalling via systemic acquired resistance (SAR)-related genes and/or an induced systematic resistance (ISR) system (Figs. 5, 6, Supplementary Fig. S4). The gene upstream of the production of the SA hormone^14^ (isochorismate synthase (*ICS*)) was significantly more highly expressed in response to 110nopE1E2 inoculation than in response to wild-type USDA110 inoculation (Fig. 5, Supplementary Fig. S4). At 4 dpi, the *GLY2* genes of KPS1, KPS2, and CN72 were also more highly expressed in response to 110nopE1E2 inoculation than in response to USDA110 inoculation, whereas the *GLY2* expression level was not significantly different between the USDA110 and 110nopE1E2 inoculation treatments at 1 dpi (Fig. 6). The *GLY2* gene encodes a G3P dehydrogenase (G3Pdh) that plays many roles in plant metabolism, but G3Pdh also enhances plant resistance^14^. The expression of gibberellin 2 oxidase (*GA2ox*) biosynthesis-related genes was induced at 1 day after USDA110 inoculation. Afterward, *GA2ox* expression drastically decreased, while gibberellin 20 oxidase (*GA20ox*) was expressed at 4 dpi. Interestingly, we also found that USDA110 can trigger plant defensin (*PDF*) gene expression in KPS2, while KPS1 *PDF* expression drastically decreased in response to USDA110 inoculation at 4 dpi (Fig. 6).

### Salicylic acid content

**Figure 7:**
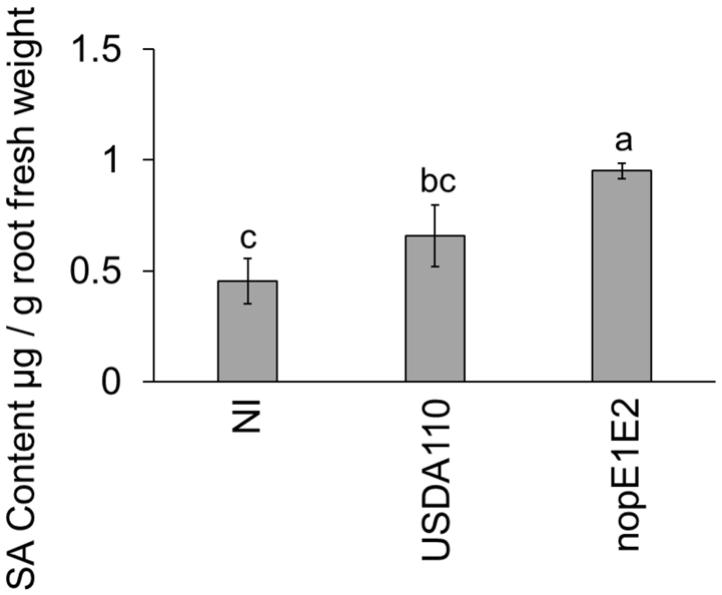
The salicylic acid content in *V. radiata* roots was determined: noninoculation (NI), USDA110 inoculation (USDA110), and double mutant strain of *nopE1* and *nopE2* (nopE1E2) inoculation. The means followed by different letters are significantly different at *P* < 0.05 is indicated by the means ± standard deviations (n = 3).

To confirm SA production, comparison of the SA contents between USDA110 and nopE1E2 inoculation at 4 dpi (Fig. 7) was employed. The results revealed that there was almost no difference in the SA content of *V. radiata* roots after NI and USDA110 inoculations. On the other hand, the SA content in the 110nopE1E2 inoculation was significantly higher than those in the NI and USDA110 inoculations. This result suggested that NopE might reduce SA accumulation at this stage of *V. radiata*-*B. diazoefficiens* USDA110 symbiosis.

## Discussion

Legume-rhizobium symbiotic interactions are controlled specifically by host and rhizobial factors and thus are not always successful or efficient. A better understanding of the molecular mechanisms underlying rhizobial factor (T3E)-triggered restriction or promotion, therefore, would provide useful insights into optimizing legume-rhizobium symbiosis and practical applications of rhizobial inoculants, especially for *V. radiata*. We further functionally characterized the type III effector NopE of *B. diazoefficiens* USDA110, which controls symbioses with different *V. radiata* varieties. It seems that USDA110 NopE is one of the factors that controls *V. radiata* symbiosis, which is highly dependent on host variety (Figs. 1, 2, 3). Almost all Thai *V. radiata* cultivars formed numerous nodules following inoculation with USDA110, whereas *V. radiata* nodulation efficiency drastically decreased when the plants were inoculated with 110T3SS and 110nopE1E2 (Table S1). In addition, nodule formation by 110nopE1E2 was not significantly different from that by 110T3SS. It seems that NopE is the key factor that promotes Thai *V. radiata*-*B*. *diazoefficiens* USDA110 symbiosis. Symbiotic functions and distinct features of NopE determining early infection and nodule organogenesis were also characterized. Finally, we further discussed how NopE is involved in different signalling pathways triggering host genotype-specific restriction or promoting nodulation among *V. radiata* varieties.

In USDA110, the *nopP* mutant had slightly reduced nodulation^15^, whereas both the *nopE1*/*nopE2* double and the T3SS-deficient mutant had strongly reduced nodulation phenotypes of KPS1 (Fig. 1), indicating that NopEs, rather than NopP, are determinants of KPS1 nodulation. Intriguingly, differences in nodulation phenotypes among the single and double *nopE* mutations suggest that NopE1 and NopE2 might yield similar results when modulating nodulation (Fig. 1). Unlike wild-type NopE1, complementation of the noncleavable NopE1 variant into the *nopE1*/*nopE2* mutant background did not enhance KPS1 nodulation (Fig. 1), highlighting the physiological role of calcium-dependent autocleavage of NopEs in promoting nodulation. Notably, the autocleavage activities of NopEs were also found to be involved in nodulation restriction in KPS2, showing that calcium-dependent autocleavage activities are strongly required for NopE functions in plant cells postsecretion and posttranslocation^7^.

**Figure 8:**
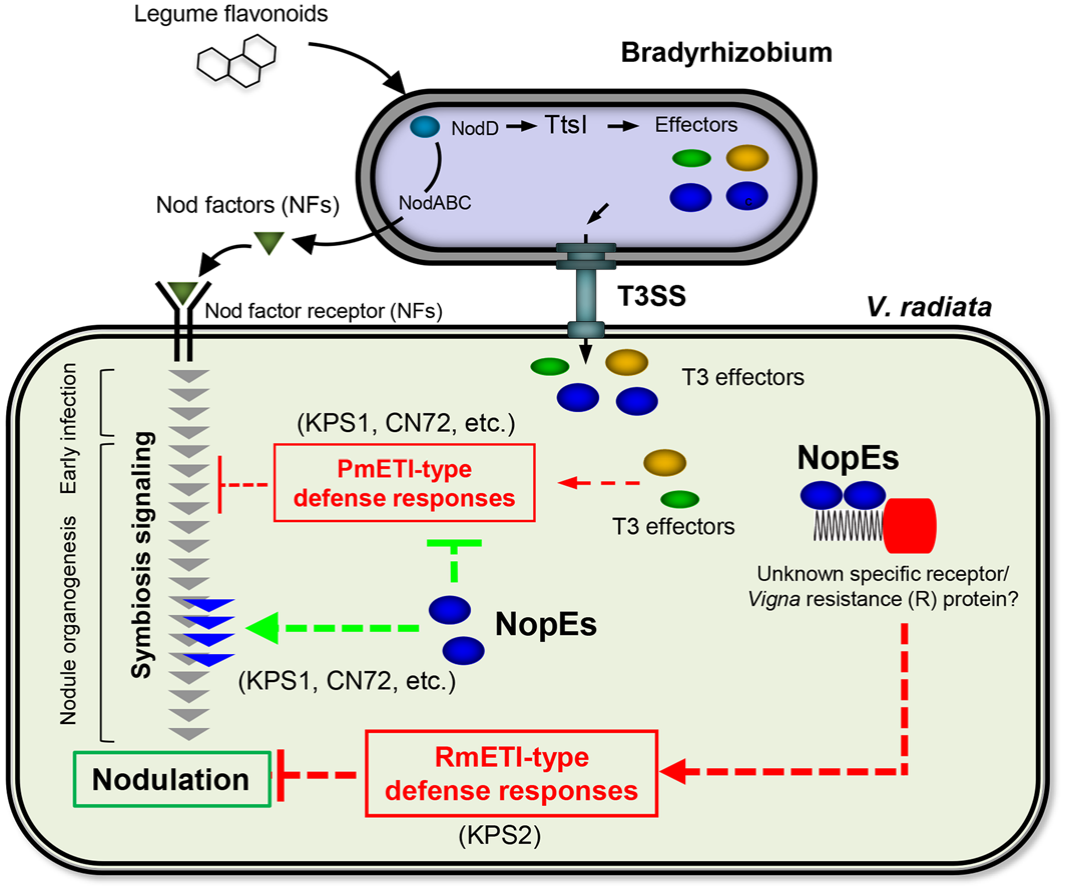
Putative models of host genotype-specific symbiotic interactions between bradyrhizobia and *V. radiata* varieties may be controlled by type III effector NopEs. Hypothetically, NopEs are nodulation determinants capable of providing the ability to nodulate *V. radiata* varieties, including KPS1, possibly by modulating downstream symbiosis signalling and/or suppressing the phytohormone-mediated effector-triggered immunity type (ETI-type) defence response (PmETI type). On the other hand, NopEs might be recognized by unknown receptor(s)/a specific resistance (R) protein in the KPS2 variety, consequently activating an R protein-mediated ETI-type defence response (RmETI-type) restricting nodulation. The dotted line presents the unclear symbiotic mechanism.

Indeed, the *nopE1*/*nopE2* double mutant of USDA110 impaired both nodule organogenesis and the formation of young nodules on taproots and in the basal regions of lateral roots of KPS1 plants (Fig. 2). Previous studies showed that T3SS and NopI of *S. fredii* HH103 were determinants for efficient nodulation of *Glycine max* (soybean) and *V. unguiculata* (cowpea); however, mutations in T3SS or NopI^16^ did not impair nodulation in these legume plant species, as NopE did for *V. radiata* (Figs. 1, 2). In *V. mungo*, inactivation of Nod factors (NFs) or T3SS/NopL of USDA61 abolished early infection and nodulation, indicating that symbiosis of *V. mungo* required both rhizobial NFs and T3Es (NopL)^6^. Notably, nodules were also formed by T3SS or *nopE1*/*nopE2* mutants, suggesting that such nodulation in *V. radiata* is likely induced via NFs in the absence of a T3SS, albeit weakly. We hypothesized that NopEs might play complementary roles in modulating symbiosis signalling and/or reducing host defence responses via unknown pathways to promote early nodulation (Fig. 8).

Notably, a few nodules occasionally formed on KPS1 inoculated with USDA61, whereas KPS2 nearly prevented nodulation by USDA110, representing different levels/types of nodulation restrictions. Similar phenomena were also observed in host-specific nodulation in soybean plants carrying a specific allele of *Rj4*, which encodes a thaumatin-like protein, and *Rj2*, which encodes a soybean R protein. In *Rj4* soybean plants, although USDA61 Bel2-5 activated phytohormone-mediated ETI-type (PmETI-type) defence responses, a few nodules were occasionally observed. Conversely, USDA122-type NopP induced R protein-mediated ETI-type (RmETI-type) defence responses in *Rj2* soybean, resulting in no or only a single nodule formed at a low frequency^15^. Taken together, our results suggest that NopEs might be recognized directly or indirectly by an unknown specific receptor or R protein in KPS2, which may be absent in KPS1 and in other *V. radiata* varieties, consequently triggering a RmETI-type defence response (Fig. 8). Further identifications of *V. radiata* genetic factors controlling rhizobial symbiosis are of interest.

NopE homologues were identified among bradyrhizobia, plant-associated endophytic bacteria and even pathogenic bacteria (Fig. S1), representing the diverse functions of NopE and its metal ion-inducible autocleavage (MIIA) activities in host-microbe interactions, including promoting symbiosis with legumes^7,17^. Unlike other rhizobium-specific T3Es, NopE homologues were not found among *Sinorhizobium* (*Ensifer*), *Rhizobium*, *Mesorhizobium*, or nearly any *B. elkanii* strain^18^, implying that rhizobia may employ NopEs and/or other T3Es as needed for promoting symbiosis with a broad range of host legumes. Nodulation-promoting functions of NopE have been observed in soybean and *Macroptilium atropurpureum*, strengthening their crucial symbiotic roles. On the other hand, several specific legumes, such as *Rj2* soybean and *V. radiata* cv. KPS2, also use these T3Es to monitor and restrict nodulation by unfavourable or inefficient rhizobial strains.

Nodule organogenesis is locally and systemically regulated by leguminous plants, and several phytohormones are induced/suppressed in response to bradyrhizobia Nod factors (NFs) and NopE secreted by *B. diazoefficiens* USDA110. In addition, legumes synthesize CLV3/ESR-related peptide signalling molecules in response to bradyrhizobial infection^14^; therefore, NopE is one of the factors that controls nodulation through phytohormone and peptide production, as described below.

A previous report showed that gibberellin 2 oxidase (GA2ox) inhibits bioactive GA in root epidermal cells. Important NF dependence and activation of GA biosynthesis-related genes suppressed DMI3 and DELLAs in the early stage of the symbiosis interaction^19^, inhibiting infection thread formation by rhizobia. In this study, the expression of GA2ox biosynthesis-related genes was induced at 1 dpi in *V. radiata* cv. KPS1 root. NopE effectors could (directly/indirectly) trigger GA2ox expression at 1 dpi, as the expression of this GA2ox catabolism-related gene was positively regulated at 1 dpi after USDA110 inoculation and downregulated at 4 dpi. Afterwards, GA20ox expression was dramatically increased at 4 dpi (Fig. 6). It seems that NopE might be one of the factors related to bioactive GA production. These results are also in agreement with previous evidence in which bioactive GA might negatively regulate infection and nodule formation^19,20^.

It is known that high-throughput sequencing tends to generate false-negative results; therefore, qRT-PCR is generally used to confirm the expression of transcript^21,22^. The data of the *CHS* had different results between qRT-PCR and NGS, it might be the false-negative of NGS result. The expression of some intermediates of SA signalling-related genes (*ICS* and *CHS*) and glycerol-3-phosphate (G3P) biosynthesis-related genes (*GLY2*) was suppressed by USDA110 NopE effectors. G3P is a mobile regulator of SAR and exhibits a broad spectrum of systemic immune responses against pathogenic invasion^14^, and incompatibility in legume-rhizobia interactions is also controlled via SAR and/or ISR systems^23^. Previous reports have shown that the restriction of symbiosis is controlled in a similar manner to that of gene-for-gene pathogenic resistance. *Rj2* and *Rfg1* are genes that encode typical Toll-interleukin receptor/nucleotide-binding site/leucine-rich repeat (TIR-NBS-LRR) receptor proteins that provide resistance to *B. japonicum* and *S. fredii* strains^11,24,25^. Interestingly, NopEs can also suppress SA biosynthesis, which governs plant defence responses (Fig. 7). However, we found that NopEs can trigger PDF expression in the incompatible *Vigna* cultivar KPS2, whereas USDA110 inoculation did not have any effect on its derived cultivar CN72 (Fig. 6). Defensins are known to be involved in plant immunity responses of the jasmonic acid (JA) pathway^12^. We propose a model modulated by USDA110 NopE in Fig. 8. The NopE effector is required for efficient nodule organogenesis by reducing PmETI via SA biosynthesis suppression, which is deleterious to rhizobial infection. Moreover, NopE also directly triggers nodulation by modulating the expression of genes that are related to the biosynthesis of plant hormones (gibberellic acid, abscisic acid, and auxin) and favour nodule organogenesis. However, nodulation likely interfered with the PDF of the JA signalling pathway in incompatible *Vigna* KPS2.

## Material and methods

### Microbiological and molecular techniques

Bradyrhizobial strains were grown at 28 °C in arabinose-gluconate (AG) medium^26^. The bacterial strains and plasmids used are summarized in Table S2. The antibiotic concentrations used were as follows (mg/L): ampicillin, 100; kanamycin, 50; and spectinomycin, 100.

### Plant materials and analyses of plant nodulation and symbiosis

A total of 20 V*. radiata* varieties were provided by Professor Piyada Alisha Tantasawat. Among the *V. radiata* tested genotypes, 13 were Thai certified/published varieties that are popularly cultivated by Thai farmers (KPS1, KPS2, SUT1, SUT2, SUT3, SUT4, SUT5, CN36, CN72, CN84-1, M4-2, M5-1, and PSU-1). The 7 other *V. radiata* genotypes were obtained from the World Vegetable Centre in Taiwan (Table S3). The study complies with local and national regulations in Thailand.

*B. diazoefficiens* USDA110 and its derivatives were grown for 5 days as previously described and used as inocula. The *V. radiata* varieties used in this study are listed in Table S3. The *V. radiata* seeds used were sterilized and subsequently germinated^8^. The seeds were sown in Leonard’s jars filled with sterilized vermiculite. The plants were watered with BNM^27^ and grown under the following controlled environmental conditions: a 28 ± 2 °C temperature, a 16 h light/8 h dark photoperiod, a light intensity of 300 μE/m^2^/s and 50% relative humidity. Five days after planting, each seedling was inoculated with 1 mL of a 5-day-old inoculum (log-phase) after washing and adjusting the optical density at 600 nm to 1 (approximately 10^7^ cells/mL). The symbiotic phenotypes were evaluated at 7, 11, 15, and 35 days postinoculation (dpi) by analysing the root image, nodule section, nodule number, whole plant dry weight, and nodule primordia formation per centimetre of root tissue.

### Bacterial RNA isolation and qRT-PCR

The mid-log-phase culture of USDA110 was washed, and the OD_600_ was adjusted to approximately 0.4 with AG media supplemented with purified flavonoids (20 μM genistein dissolved in DMSO). DMSO alone was used as a negative control. Bacterial cells were cultured at 28 °C for 16 h and collected by centrifugation (10,000 × rpm for 5 min at 4 °C), after which they were immediately frozen in liquid nitrogen prior to storage at − 80 °C for further total RNA isolation.

Total bacterial RNA was extracted from induced cells using an RNeasy Mini Kit (QIAGEN, United States) according to the manufacturer’s protocol. Total RNA was treated with RNase-free DNase I (NEB) for 30 min at 37 °C, and DNA contamination was determined based on PCR amplification using a primer pair specific to the endogenous housekeeping gene (16S rRNA). cDNA was then synthesized from 500 ng of total RNA (without DNA contamination) using a High-Capacity cDNA Reverse Transcription Kit (iScript, Bio-Rad) according to the manufacturer’s protocol. Twenty-five nanograms of cDNA was subjected to PCR amplification using gene-specific primers (Table S4). PCR amplification was performed using QuantStudio 3 Real-Time PCR System Mix (Applied Biosystems) in conjunction with the following PCR program: an initial denaturation step at 95 °C for 2 min; 35 cycles of 95 °C for 30 s, 55 °C for 30 s, and 72 °C for 15 s; and a final extension step at 72 °C for 10 min. The relative gene expression was calculated using the comparative Ct (-ΔΔCT) method^28^ and normalized to the expression of 16S rRNA using the primers PBA338F (5′-ACTCCTACGGGAGGCAGCAG-3′) and PRUN518R (5′-ATTACCGCGGCTGCTGG-3′). The data from three biological replicates were pooled and analysed. At least three PCR amplifications were performed for each sample.

qRT-PCR of selected DEGs was performed using the same protocol as that used for bacterial gene expression quantification. The primer sets used for qRT-PCR are listed in Table S4. The transcript levels of selected *V. radiata* DEGs were normalized against the expression of its *β-actin* housekeeping gene^29^ measured in the same samples.

### Cytological analysis of nodules

Fresh nodules were examined under a Leica Microsystems 10447197 EZ4 Stereo Microscope (Leica Nanterre, France). Sections (40–50 μm thick) of fresh nodules were prepared using a VT1000S vibratome (Leica Nanterre, France) and then observed under a compound microscope (Carl Zeiss, Germany). Confocal images were taken with a Nikon A1Rsi inverted confocal microscope (Nikon, USA). Nodule sections were incubated for 15 min in live/dead staining solution (5 μM SYTO 9 and 30 μM propidium iodide in 50 mM Tris pH 7.0 buffer; Live/Dead BacLight, Invitrogen, Carlsbad, CA, USA), nodule sections were then stained for an additional 20 min with calcofluor white M2R (0.01% (wt/vol) calcofluor white M2R in 10 mM phosphate saline buffer) (Sigma, Munich, Germany) to observe the plant cell wall^30^. Calcofluor white was excited at 405 nm and detected via a 460–500 nm emission filter. For SYTO 9 and propidium iodide, excitation wavelengths of 488 and 555 nm were used to collect emission signals at 490–522 nm and 555–700 nm, respectively.

### *V. radiata* mRNA transcriptome analysis

For RNA-seq of *V. radiata* roots, seeds were surface sterilized, germinated at 25 °C for 2 days, and then transplanted into Leonard’s jars together with BMN nitrogen-free solution. Five days after planting, the plants were inoculated with 2 × 10^7^ cells/mL bacterial cultures (3 biological replicates for each sample). Afterwards, at 4 dpi, the *V. radiata* roots were immediately frozen in liquid nitrogen and then ground to a fine powder; 100 mg of the powder was used for total RNA extraction using an RNeasy Plant Mini Kit (Qiagen) and treatment with DNase I (Qiagen) according to the manufacturers’ instructions. A cDNA library was constructed from 4 μg of total RNA following the manufacturer’s protocol of a TruSeq Stranded mRNA LT Sample Prep Kit (Illumina). The sequence of each library using the Illumina platform and a bioinformatic approach were determined by GENEWIZ Suzhou, China. The differentially expressed genes (DEGs) were selected for further analysis based on *P*adj < 0.05 and fold change > 2.

### Measurements of SA content

For salicylic acid quantification of *V. radiata* roots, seeds were surface sterilized, germinated at 25 °C for 2 days, and then transplanted into Leonard’s jars together with BMN nitrogen-free solution. Five days after planting, the plants were inoculated with 2 × 10^7^ cells/mL bacterial cultures. Afterwards, at 4 dpi, the *V. radiata* roots were collected and ground into a fine powder in liquid nitrogen; 100 mg of the powder was used, to which 1 mL of 90% methanol was added. The mixtures were homogenized using a blender for 2 min at 6000 rpm and then soaked overnight at 4 °C. The mixtures were then centrifuged at 1204×*g* for 10 min at 4 °C. The supernatants were collected and evaporated to dryness with a gentle stream of nitrogen and then resuspended in 20 µL of trichloroacetic acid (1 M). The mixtures were partitioned against cyclohexane/ethyl acetate (1:1) three times, and the organic phase was collected and dried in nitrogen. The dried organic phase of the root extract was reconstituted with 500 µL of methanol and filtered through a 0.22 µm PTFE membrane prior to high-performance liquid chromatography-DAD detector (HPLC–DAD) analysis. The separations were performed on a Hypersil GOLD C18 column (100 mm × 2.1 mm ID, 1.9 µm) (Thermo Scientific, USA) at 30 °C.

### Statistical analysis

For phylogenetic and evolutionary analyses, NopE homologues were queried via BLASTx against genomic databases of bacterial species, including rhizobial, endophytic, and pathogenic bacteria. A phylogenetic tree was constructed using the neighbour-joining method based on the Poisson model of the MEGA 7.0 package, with 1000 bootstrap replications. For statistical analyses, one-way analysis of variance (ANOVA) followed by post hoc tests (Tukey’s tests at *P* ≤ 0.05) was performed using Minitab version 16.0 for multiple test sample comparisons. Two-tailed Student’s *t*-tests were also performed for pairwise comparisons when needed. *P*-values < 0.05 were considered statistically significant. The sample size and replications are detailed in the figure and table legend.

## Supplementary Information

Supplementary Information.

## References

[1] Oldroyd GED (2013). Speak, friend, and enter: Signalling systems that promote beneficial symbiotic associations in plants. Nat. Rev. Microbiol..

[2] Krause A, Doerfel A, Göttfert M (2002). Mutational and transcriptional analysis of the type III secretion system of *Bradyrhizobium japonicum*. Mol. Plant-Microbe Interact..

[3] Miwa H, Okazaki S (2017). How effectors promote beneficial interactions. Curr. Opin. Plant Biol..

[4] Staehelin C, Krishnan HB (2015). Nodulation outer proteins: Double-edged swords of symbiotic rhizobia. Biochem. J..

[5] Okazaki S, Zehner S, Hempel J, Lang K, Göttfert M (2009). Genetic organization and functional analysis of the type III secretion system of *Bradyrhizobium elkanii*. FEMS Microbiol. Lett..

[6] Nguyen HP, Ratu STN, Yasuda M, Teaumroong N, Okazaki S (2020). Identification of *Bradyrhizobium elkanii* USDA61 type III effectors determining symbiosis with *Vigna mungo*. Genes (Basel).

[7] Wenzel M, Friedrich L, Göttfert M, Zehner S (2010). The type III-secreted protein NopEl affects symbiosis and exhibits a calcium-dependent autocleavage activity. Mol. Plant-Microbe Interact..

[8] Piromyou P (2019). Mutualistic co-evolution of T3SSs during the establishment of symbiotic relationships between *Vigna radiata* and Bradyrhizobia. Microbiologyopen..

[9] Ngampongsai S (2008). Current status of mungbean and the use of mutation breeding in Thailand. F. Crop. Res..

[10] Dao TTH, Linthorst HJM, Verpoorte R (2011). Chalcone synthase and its functions in plant resistance. Phytochem. Rev..

[11] Hayashi M (2014). A thaumatin-like protein, Rj4, controls nodule symbiotic specificity in soybean. Plant Cell Physiol..

[12] Serrazina S, Machado H, Costa RL, Duque P (2021). Expression of *Castanea crenata* Allene Oxide Synthase in Arabidopsis Improves the Defense to *Phytophthora cinnamomi*. Front. Plant Sci..

[13] Boivin S, Fonouni-Farde C, Frugier F (2016). How auxin and cytokinin phytohormones modulate root microbe interactions. Front. Plant Sci..

[14] Shine MB (2019). Glycerol-3-phosphate mediates rhizobia-induced systemic signaling in soybean. Nat. Commun..

[15] Sugawara M (2018). Variation in bradyrhizobial NopP effector. Nat. Commun..

[16] Jiménez-Guerrero I, Pérez-Montaño F, Medina C, Ollero FJ, López-Baena FJ (2017). The *Sinorhizobium* (*Ensifer*) *fredii* HH103 nodulation outer protein NopI is a determinant for efficient nodulation of soybean and cowpea plants. Appl. Environ. Microbiol..

[17] Hoyer E (2019). Calcium binding to a disordered domain of a type III-secreted protein from a coral pathogen promotes secondary structure formation and catalytic activity. Sci. Rep..

[18] Imperial J, Dur D, Zehner S, Rey L, Michael G (2018). Characterization of a novel MIIA domain-containing protein (MdcE) in *Bradyrhizobium* spp.. FEMS Microbiol. Lett..

[19] Buhian WP, Bensmihen S (2018). Mini-review: Nod factor regulation of phytohormone signaling and homeostasis during rhizobia-legume symbiosis. Front. Plant Sci..

[20] Kim GB, Son SU, Yu HJ, Mun JH (2019). MtGA2ox10 encoding C20-GA2-oxidase regulates rhizobial infection and nodule development in *Medicago truncatula*. Sci. Rep..

[21] Boonchuen P, Jaree P, Somboonviwat K, Somboonwiwat K (2021). Regulation of shrimp prophenoloxidase activating system by lva-miR-4850 during bacterial infection. Sci. Rep..

[22] Ladetto M (2014). Next-generation sequencing and real-time quantitative PCR for minimal residual disease detection in B-cell disorders. Leukemia.

[23] Wang Q, Liu J, Zhu H, Harris JM (2018). Genetic and molecular mechanisms underlying symbiotic specificity in legume-*Rhizobium* interactions. Front. Plant Sci..

[24] Yang S, Tang F, Gao M, Krishnan HB, Zhu H (2010). R gene-controlled host speci fi city in the legume—Rhizobia symbiosis. Proc. Natl. Acad. Sci. U.S.A..

[25] Fan Y (2017). The soybean Rfg1 gene restricts nodulation by *Sinorhizobium fredii*. Front. Plant Sci..

[26] Sadowsky MJ, Tully RE, Cregan PB, Keyser HH (1987). Genetic diversity in *Bradyrhizobium japonicum* serogroup 123 and its relation to genotype-specific nodulation of soybean. Appl. Environ. Microbiol..

[27] Ehrhardt DW, Morrey Atkinson E, Long SR (1992). Depolarization of alfalfa root hair membrane potential by *Rhizobium meliloti* nod factors. Science.

[28] Livak KJ, Schmittgen TD (2001). Analysis of relative gene expression data using real-time quantitative PCR and the 2-ΔΔCT method. Methods.

[29] Bjarnadottir H, Jonsson JJ (2005). A rapid real-time qRT-PCR assay for ovine β-actin mRNA. J. Biotechnol..

[30] Nagata T, Takebe I (1970). Cell wall regeneration and cell division in isolated tobacco mesophyll protoplasts. Planta.

