# Supplementary material for: The Bradyrhizobium diazoefficiens type III effector NopE modulates the regulation of plant hormones towards nodulation in Vigna radiata
Source: Sci Rep. 2021 Aug 16;11:16604. doi: 10.1038/s41598-021-95925-4 (PMC8367979; doi:10.1038/s41598-021-95925-4)
Supplement: Supplementary file 1 — Supplementary Information. [file 41598_2021_95925_MOESM1_ESM.pdf]

Supplementary materials

The *Bradyrhizobium diazoefficiens* type III effector NopE modulates the regulation of plant hormones towards nodulation in *Vigna radiata*

Pongdet Piromyou[1, †] and Hien P. Nguyen[2, 7, †], Pongpan Songwattana[1], Pakpoom Boonchuen[1], Kamonluck Teamtisong[3], Panlada Tittabutr[1], Nantakorn Boonkerd[1], Piyada Alisha Tantasawat[4], Michael Göttfert[5], Shin Okazaki[6, *], Neung Teaumroong[1, *]

1 School of Biotechnology, Institute of Agricultural Technology, Suranaree University of Technology, Nakhon Ratchasima, 30000, Thailand

2 Institute of Global Innovation Research (IGIR), Tokyo University of Agriculture and Technology (TUAT), Fuchu, Tokyo 183-8538, Japan

3 The Center for Scientific and Technological Equipment, Suranaree University of Technology, Nakhon Ratchasima 30000, Thailand

4 School of Crop Production Technology, Institute of Agricultural Technology, Suranaree University of Technology, Nakhon Ratchasima 30000, Thailand

5 Technische Universität Dresden, Institut für Genetik, Helmholtzstrasse 10, 01062 Dresden, Germany

6 Graduate School of Agriculture, TUAT, Fuchu, Tokyo 183-8509, Japan

7 Beltsville Agricultural Research Center (BARC), Agricultural Research Service (ARS), the U.S. Department of Agriculture (USDA), Beltsville, MD 20705, USA

† These authors contributed equally as co-first authors: Pongdet Piromyou and Hien P. Nguyen

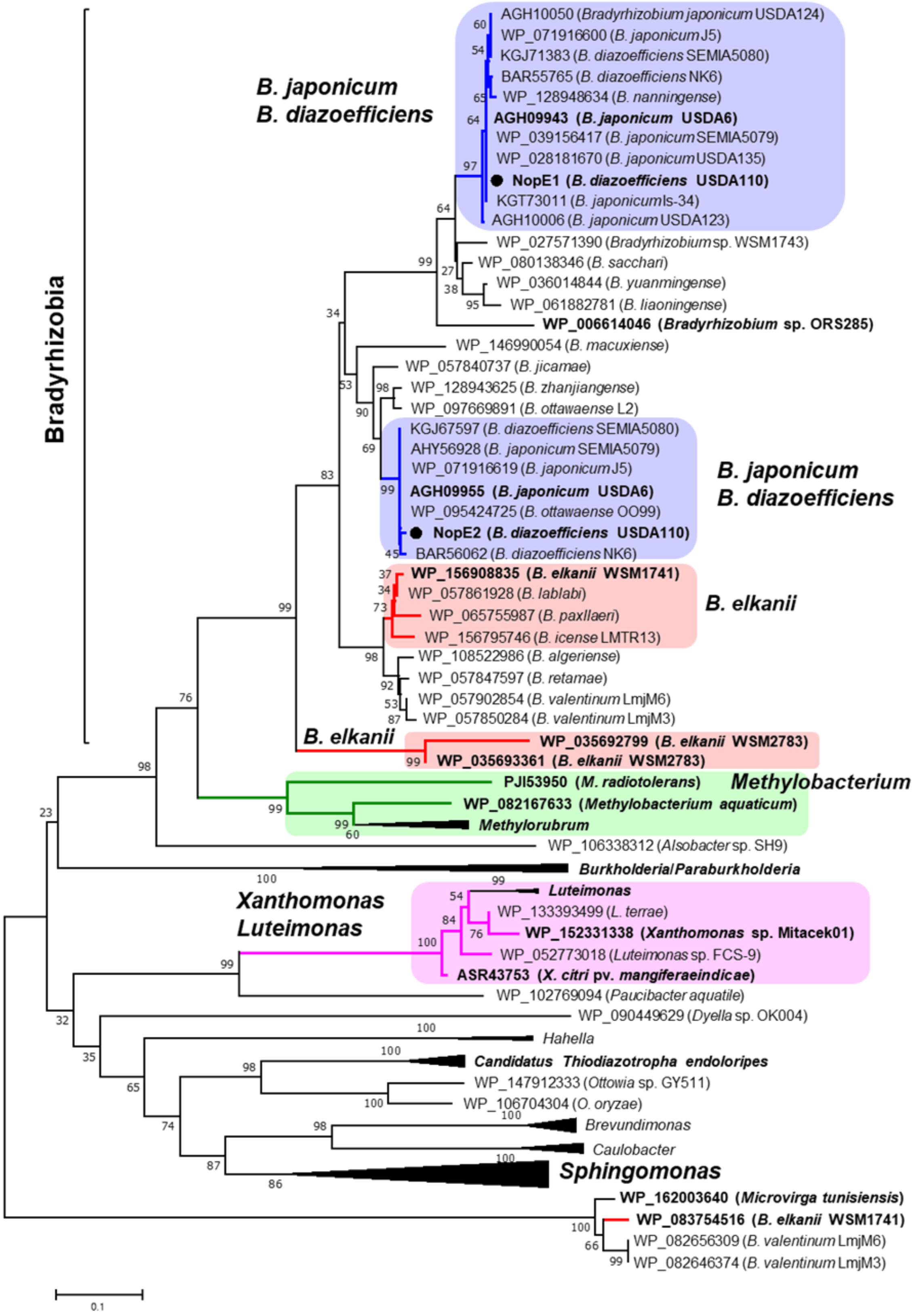

Figure S1 Phylogenetic analysis of *Bradyrhizobium diazoefficiens* USDA110 NopEs and related proteins. Bootstrap values are expressed as percentages of 10,000 replications. The NopEs of *B. elkanii* (red), *B. japonicum*/*B. diazoefficiens* (blue), *Microvirga* (green), and *Xanthomonas* (pink) groups are highlighted. No homologs of NopEs could be identified in *Sinorhizobium* (*Ensifer*) spp., *Rhizobium* spp., and *Mesorhizobium* spp.

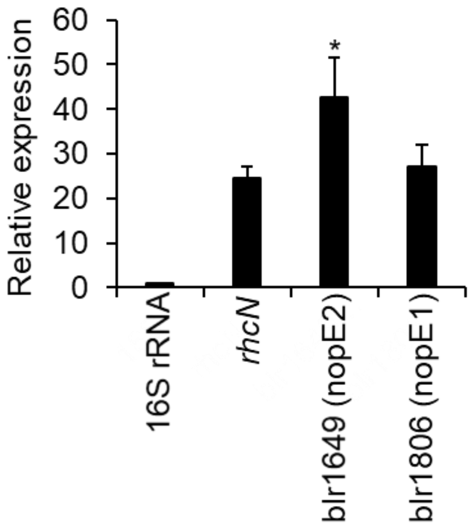

Figure S2 The relative expression of 16S rRNA, *rhcN*, and putative T3Es genes (*nopE1* and *nopE2*) of *B. diazoefficiens* USDA110 after induction with genistein. The level of expression was measured using qRT-PCR. Values represent mean ± SD (n = 3), "*", $P < 0.05$ according to Student's *t*-test.

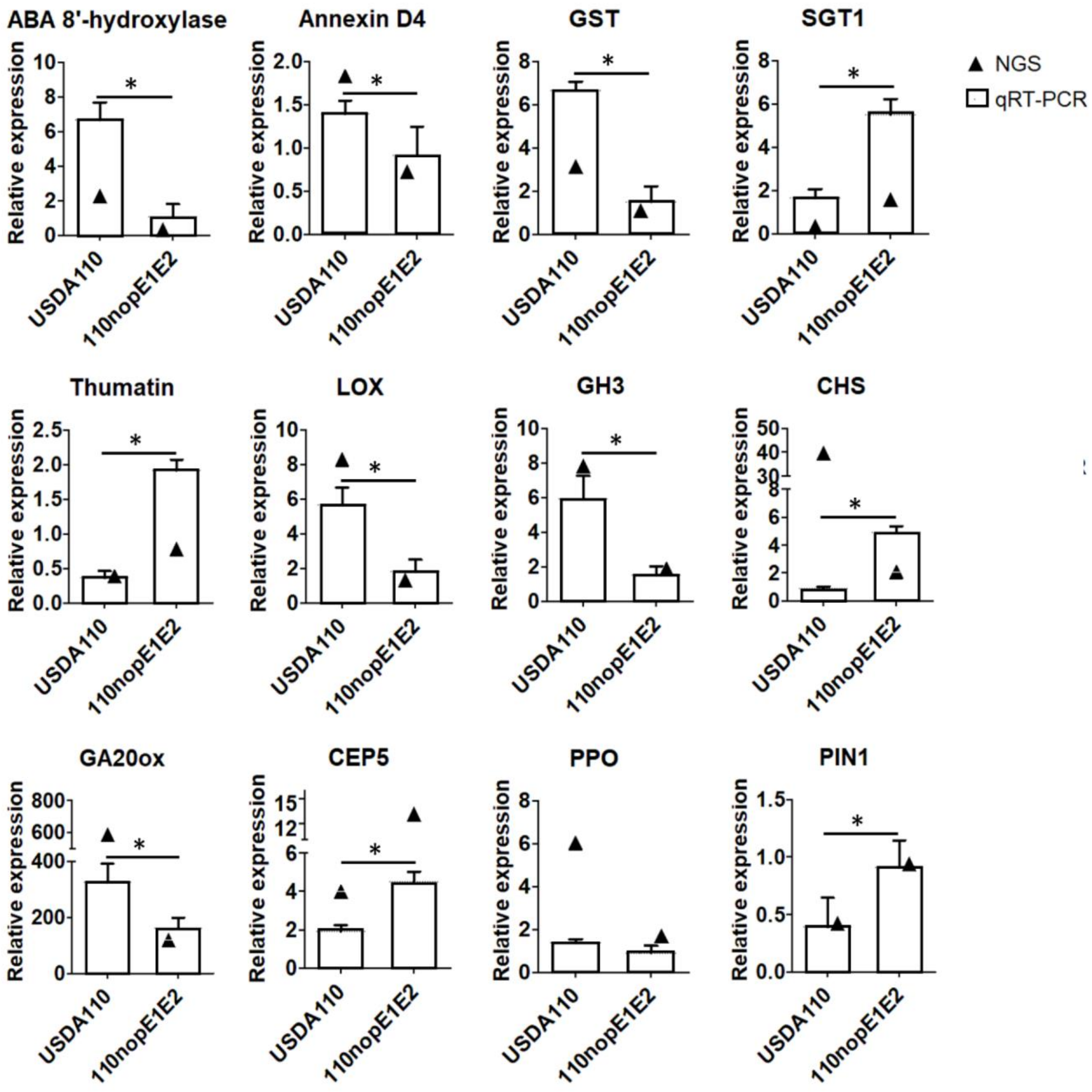

Figure S3 qRT-PCR verification from RNA-seq analysis data for *Vigna radiata* cv. KPS1 between USDA110 and 110nopE1E2 inoculations at and 4 dpi. Significance is indicated by the mean±standard deviation (n=3), "*", $P < 0.05$ according to Student's *t*-test.

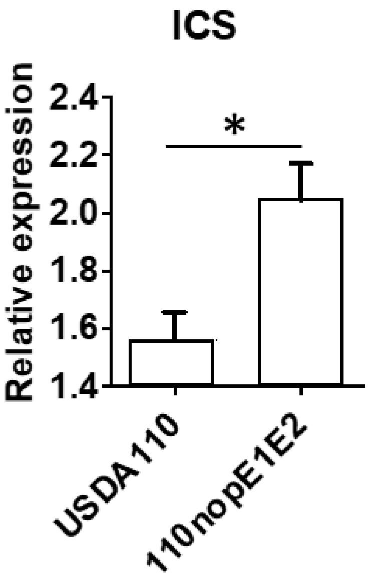

Figure S4 qRT-PCR analysis data for *Vigna radiata* cv. KPS1 between USDA110 and 110nopE1E2 inoculations at and 4 dpi. Significance is indicated by the mean±standard deviation (n=3), “*”, $P < 0.05$ according to Student’s *t*-test.

Table S1 Symbiotic phenotypes of *Vigna radiata* varieties inoculated with bradyrhizobial strains.

V. radiata variety	*Bradyrhizobium diazoefficiens**			
	USDA110 or 110spc4	110ttsI	110T3SS	110nopE1E2
V. radiata var. *radiata* (Thai cultivated cultivars)				
KPS1	+++	+	+	+
KPS2	–	+++	+++	+++
SUT1	+++	+	+	+
SUT2	+++	+	+	+
SUT3	+++	+	+	+
SUT4	+++	+	+	+
SUT5	+++	+	+	+
CN36	+++	+	+	+
CN72	+++	+	+	+
CN84-1	+++	+	+	+
M4-2	+++	+	+	+
M5-1	+++	+	+	+
PSU-1	+++	+	+	+
V. radiata var. *radiata* (From Asian Vegetable Research and Development Centre: AVRDC)				
V3092	++	+	+	+
V3131	++	+	+	+
V3387	+	++	++	++
V3464	++	+	+	+
V4451	+	++	++	++
V5197	+	++	++	++

+, efficient nodulation. The number of "+" indicate levels of nodulation efficiency.

*, Nodule formation was statistically difference between wild type and T3SS mutant strain.

Table S2 Bacterial strains used in this study.

Strains	Characteristics[a]	Reference
Bradyrhizobium diazoefficiens		
USDA110	Wild-type strain, Pol^r	USDA[b]
110spc4	A spectinomycin-resistant derivative of *B. diazoefficiens* USDA110, Sp^r	(Regensburger and Hennecke, 1983)
110T3SS	Δ(*nolB*, *rhcJ*, *nolU*, *nolV*, and *rhcN*), previously designated as Δ132 mutant derived from *B. diazoefficiens* 110spc4, Km^r, Sp^r	(Wenzel et al., 2010)
110nopE1	*nopE1* replaced by *aadA* from pHP45Ω, previously designated as BJDΔ718 mutant, Sp^r, Sm^r	(Wenzel et al., 2010)
110nopE2	Disruption of *nopE2* by cointegration of plasmid pBJD215, previously designated as BJD215 mutant, Tc^r	(Wenzel et al., 2010)
110nopE1E2	*nopE1* and *nopE2* deletion mutant, previously designated as BJDΔ718-Δ644 mutant, Sp^r, Sm^r, Km^r	(Wenzel et al., 2010)
110E1E2::E1	110E1E2 derivative complemented with the wild-type *nopE1*, previously designated as BJD735, Sp^r, Sm^r, Km^r, Tc^r	(Wenzel et al., 2010)
110E1E2::ncE1	110E1E2 derivative complemented with the non-cleavable *nopE1* variant, previously designated as BJD740, Sp^r, Sm^r, Km^r, Tc^r	(Wenzel et al., 2010)

[a]Pol^r, polymyxin resistant; Km^r, kanamycin resistant; Sm^r, streptomycin resistant; Sp^r, spectinomycin resistant; Tc^r, tetracycline resistant.

[b]United States Department of Agriculture, Beltsville, MD.

Table S3 Pedigree, origin and relevant characteristics

Genotype	Pedigree	Origin	Source or reference
KPS1	Mass selection from VC1973A (V3476 x V1400)	Thailand	SUT [1, (a)]
KPS2	Mass selection from VC2778A	Thailand	SUT [1, (a)]
SUT1	UTHONG1 x NP29	Thailand	SUT [2, (b)]
SUT2	-	Thailand	SUT [2, (b)]
SUT3	-	Thailand	SUT [2, (b)]
SUT4	MV1 x (MX4-7MO317 x MO277)	Thailand	SUT [2, (b)]
SUT5	-	Thailand	SUT [2, (b)]
CN36	PAGASA1 x PHLV18	Thailand	SUT [1, (c)]
CN72	Selection from mutated KPS2	Thailand	SUT [3, (c)]
CN84-1	Selection from mutated CN36	Thailand	SUT [4, (c)]
M4-2	Selection from mutated KPS1	Thailand	SUT [5, (a)]
M5-1	Selection from mutated KPS1	Thailand	SUT [5, (a)]
PSU-1	Mass selection from VC2768A	Thailand	SUT [1, (d)]
V3092	-	India	AVRDC [(e)]
V3131	-	-	AVRDC [(e)]
V3387	-	-	AVRDC [(e)]
V3464	-	-	AVRDC [(e)]
V4451	-	-	AVRDC [(e)]
V4785	-	India	AVRDC [(e)]
V5197	-	-	AVRDC [(e)]

- = unknown data

SUT = Seed was provided by Professor Piyada Alisha Tantasawat, Suranaree University of Technology, Thailand

AVRDC = Seed was provided by Asian Vegetable Research and Development Center/ World Vegetable Center, Taiwan

[(a)] = Varieties from Kasetsart University, Kamphaeng Saen Campus, Thailand

[(b)] = Varieties developed at Suranaree University of Technology, Thailand

[(c)] = Varieties developed at Chai Nat Field Crops Research Center, Thailand

[(d)] = Varity from Prince of Songkla University, Thailand

[(e)] = Varieties obtained from the World Vegetable Center, Taiwan

Table S4 Primers used in this study

Target	Primer name	Primer sequence (5’→3’)
16S rRNA	PBA338F	ACTCCTACGGGAGGCAGCAG
	PRUN518R	ATTACCGCGGCTGCTGG
rhcN	U110-rhcNf	CCGCGTGATCGACAGTTTTG
	U110-rhcNr	GCGCCGTTTCATCGGATTAG
blr1649 (*nopE2*)	U110-blr1649f	ATATACGATCACAGCCAATG
	U110-blr1649r	ATCCTTGCCGTCCCCGTTAG
blr1806 (*nopE1*)	U110-blr1806f	CTACCAGTACTTGCCGGTAG
	U110-blr1806r	ATGTACGCCTGCGTCGTAAC
Vigna radiata: actin-3	VrActin.f	CAGTGTCTGGATTGGAGGCT
	VrActin.r	GTCCTCGACCACTTGATG
Vigna radiata: NDR1	Vir-NDR1.F	TTTACGTTCCTGCCCTCAAC
	Vir-NDR1.R	GAGGTGAACGTCGTCGTATTT
Vigna radiata: PAR1	Vir-Par1.F	CTGCCAAGGAAGGTAATGAAGA
	Vir-Par1.R	GCCAGAATATAGCCAGCAGTT
Vigna radiata: EDS1	Vir-EDS1L.F	GGTATGGGATGAGGTGATTGAG
	Vir-EDS1L.R	CCGATGGTAGTTGGCTATGTC
Vigna radiata: PTi5	PTI5-Vr.f	CTCACCAAAGACTCCACCAA
	PTI5-Vr.r	ACGATTTCGGCTGGGAAA
Vigna radiata: PTi6	PTI6-Vr.f	CTTTAACCCGACCCGAGAAG
	PTI6-Vr.r	GCGAAACGTGGAGTTCTCT
Vigna radiata: PR1	PR1-Vr.f	CGAATGGACCTTATGGTGAGAA
	PR1-Vr.r	TTACCAGGAGGGTCGTAGTT
Vigna radiata: PR2	PR2-Vr.f	CCCAAAGGCTGTTGATTCTTTC
	PR2-Vr.r	CGTTGAGGCCATCACTCAATA
Vigna radiata: PR4	PR4-Vr.f	ATAATGTCCGGGCCACATATC
	PR4-Vr.r	ACAATCCTCACCGTTGTCTG
Vigna radiata: PR5	PR5-Vr.f	CGCTCAACCCGACTTCTAC
	PR5-Vr.r	AGTTCCCTCTGCAGCAATAC
Vigna radiata: ICS	Vir-ICS2.F	CACTAGGGTCGTACCTACTTCT
	Vir-ICS2.R	TAGTTGCTCTGGTGTGTTTCC
Vigna radiata: GLY2	Vir-GLY2.F	GCTTTGCTTGGTGCTGATTAC
	Vir-GLY2.R	TTGGCGAGGATTTCGTAGTG
Vigna radiata: CHS	Chalcone.exp.f	ACCCCAACATAAGCACTTAC
	Chalcone.exp.r	AGCCTGACAAGTTGGTAATC
Vigna radiata: PDF	Vr-defensin.exp.f	GAGGCAAAGACTTGCGAGAAC
	Vr-defensin.exp.r	TTCGGGTTGGGGAGAATCCGGTT
Vigna radiata: GA2ox	GA-2.exp.f	CGTCAACCACGGTGTTCCATTG
	GA-2.exp.r	CCAACCGACATCGCCGTTTG
Vigna radiata: GA20ox	GA-20.exp.f	AGAAGTACTGCGATGGTATG
	GA-20.exp.r	CTGTCCCCAACGTAAGGTTC
Vigna radiata: ABA 8’-hydrolase	ABA 8’-hydrolase.exp.f	CAAGAGAAAGGAGCAGAGATTG
	ABA 8’-hydrolase.exp.r	GTAGTATCCTGAGCTGCAAAC
Vigna radiata: Annex D4	Annexin.exp.f	AGGCAGTTTACAAGCACTAC
	Annexin.exp.r	ATCAATTCTCAATGCTGCATTC
Vigna radiata: GST	PeroxidaseA2.exp.f	GTTGCGATGGATCAGTTTTG
	PeroxidaseA2.exp.r	AAGCTACTTCAGCTGCAATG
Vigna radiata: SGT1	SGT1.exp.f	GCAAGTTCCCATCACACAAAG
	SDT1.exp.r	CAATCAGCTTCGTGGATGTTG
Vigna radiata: Thumatin	Thaumatin-exp.f	TCTCCCTGGTTGATGGTTTC
	Thaumatin-exp.r	CGTCTTAAACACAGTGCATG
Vigna radiata: LOX	LOX.exp.f	CTTCCAAGGGATGAATCATTTG
	LOX.exp.r	GTCCACGCACATCATCAAAG
Vigna radiata: GH3	GH3.exp.f	AAAGGCAAGGGTATGTACTTTC
	GH3.exp.r	AATGGGGCTGGTGTAGTTTG
Vigna radiata: CEP5	CEP5.exp.f	AGGAGCACGTGATGGGAATG
	CEP5.exp.r	CAGGATCCGTTGGTCGGAAATC

Vigna radiata: PPO	PPO.exp.f	GATGGTGCATATCACCAAGTTG
	PPO.exp.r	GGAGCATCCCAGTTCCAAAATG
Vigna radiata: PIN1	PIN.exp.f	ACAGAGGCGCCAGAATGTTG
	PIN.exp.r	TGACGGTGACGTGGAGCTTTC

References

1. Regensburger, B. & Hennecke, H. RNA polymerase from *Rhizobium japonicum*. *Arch. Microbiol*. **135**, 103–109 (1983).
2. Wenzel, M., Friedrich, L., Göttfert, M. & Zehner, S. The type III-secreted protein NopE1 affects symbiosis and exhibits a calcium-dependent autocleavage activity. *Mol. Plant-Microbe Interact*. **23**, 124–129 (2010).
3. Somta, P., Sommanas, W. & Srinives, P. Molecular diversity assessment of AVRDC-The World Vegetable Center elite-parental mungbeans. *Breed. Sci*. **59**, 149–157 (2009).
4. Tantasawat, P. A., Khajudparn, P., Prajongjai, T. & Poolsawat, O. Heterosis for the improvement of yield in mungbean [*Vigna radiata* (L.) wilczek]. *Genet. Mol. Res*. **14**, 10444–10451 (2015).
5. Ngampongsai, S. et al. Current Status of Mungbean and the Use of Mutation Breeding in Thailand. *F. Crop. Res*. **1**, 1–6 (2008).
6. View of Genetic relationship of mungbean and blackgram genotypes based on agronomic and photosynthetic performance and SRAP markers.pdf.
7. View of Mutant Mungbean Lines from Radiation and Chemical Induction.pdf
